# RBD-homodimer, a COVID-19 subunit vaccine candidate, elicits immunogenicity and protection in rodents and nonhuman primates

**DOI:** 10.1038/s41421-021-00320-y

**Published:** 2021-09-07

**Authors:** Xiaoyan Pan, Jian Shi, Xue Hu, Yan Wu, Liang Zeng, Yanfeng Yao, Weijuan Shang, Kunpeng Liu, Ge Gao, Weiwei Guo, Yun Peng, Shaohong Chen, Xiaoxiao Gao, Cheng Peng, Juhong Rao, Jiaxuan Zhao, Cheng Gong, Hui Zhou, Yudong Lu, Zili Wang, Xiliang Hu, WenJuan Cong, Lijuan Fang, Yongxiang Yan, Jing Zhang, Hui Xiong, Jizu Yi, Zhiming Yuan, Pengfei Zhou, Chao Shan, Gengfu Xiao

**Affiliations:** 1 State Key Laboratory of Virology, Wuhan Institute of Virology, Chinese Academy of Sciences, Wuhan, Hubei China; 2 University of the Chinese Academy of Sciences, Beijing, China; 3 Center for Biosafety Mega-Science, Wuhan Institute of Virology, Chinese Academy of Sciences, Wuhan, Hubei China; 4 Wuhan YZY Biopharma Co., Ltd., Wuhan, Hubei China

**Keywords:** Immunology, Biological techniques

## Abstract

The pandemic of COVID-19 caused by SARS-CoV-2 has raised a new challenges to the scientific and industrious fields after over 1-year spread across different countries. The ultimate approach to end the pandemic is the timely application of vaccines to achieve herd immunity. Here, a novel SARS-CoV-2 receptor-binding domain (RBD) homodimer was developed as a SARS-CoV-2 vaccine candidate. Formulated with aluminum adjuvant, RBD dimer elicited strong immune response in both rodents and non-human primates, and protected mice from SARS-CoV-2 challenge with significantly reducing viral load and alleviating pathological injury in the lung. In the non-human primates, the vaccine could prevent majority of the animals from SARS-CoV-2 infection in the respiratory tract and reduce lung damage. In addition, antibodies elicited by this vaccine candidate showed cross-neutralization activities to SARS-CoV-2 variants. Furthermore, with our expression system, we provided a high-yield RBD homodimer vaccine without additional biosafety or special transport device supports. Thus, it may serve as a safe, effective, and low-cost SARS-CoV-2 vaccine candidate.

## Introduction

Up to July, 2021, the coronavirus disease 2019 (COVID-19) pandemic caused by severe acute respiratory syndrome coronavirus 2 (SARS-CoV-2) has led to over 189 million infections and more than 4.0 million deaths, with an average fatality rate of 2.1%. The pandemic, which has lasted for more than a year, has a huge impact on public health, global economy, and cultural exchanges. Thus, it is urgent to develop rapid viral detection technologies, therapeutics, and vaccines, to stop the relentless spread of COVID-19, and effective vaccines are widely considered to be the best approach to end the pandemic^1^. At the beginning of the outbreak, multiple forms of vaccines development were conducted worldwide, including inactivated vaccine, live attenuated vaccine, viral vector vaccine, nucleic acid vaccine, and protein subunit vaccine, etc. Currently, about 300 vaccines are in clinical trials with active or completed state (http://clinicaltrials.gov). Among them, at least 26 vaccines have been approved for emergency use in several countries after or at phase III clinical trials^2^, including the vaccines that have received high attention: inactivated vaccines by Sinopharm^3,4^ and SINOVAC^5^, mRNA vaccines by BioNTech/Pfizer^6^ and Moderna^7^, adenovirus viral vector vaccines by AstraZeneca/University of Oxford^8^ and CanSino^9^, and protein subunit vaccine by Zhifei Longcom^10^ and Novavax^11^. Yet, the yields of these vaccines are still too low to meet the worldwide needs. In spite of that, the production process of the nucleic acid vaccine or protein-based vaccine is not restricted by the biosafety facility, and thus can be expected to provide a massive product in a short term to meet the markets’ demands, while mRNA vaccine needs neither –20 °C nor –80 °C for the storage and transport, which may limit its use in developing countries.

As it is well known that SARS-CoV-2 belongs to the beta coronavirus, the virus is the seventh jump to mankind from wildlife animals or environments, other than HCoV-229E, HCoV-OC43, HCoV-NL63, HCoV-HKU1, SARS-CoV, and Middle East Respiratory Syndrome (MERS)-CoV^12^. Upon its identification as the pathogen of COVID-19^13^, the viral characteristics and its host receptor were quickly decoded^14^, meanwhile, the pathologies caused by SARS-CoV-2 infection were successively verified in transgenic mice^15^ and non-human primates^16^. The Spike protein (S) of SARS-CoV-2, which decorates on the surface of the spherical virion particles, recognizes the host receptor of human cells, angiotensin converting enzyme II (ACE2), and mediates the viral invasion process. The core of S protein binding to human ACE2 (hACE2) is identified as 319–541 amino acid region which was defined as receptor-binding domain (RBD)^17^. Therefore, the RBD is certainly considered a candidate for SARS-CoV-2 vaccine development^18^. As a matter of fact, RBD monomer^19^, tandem RBD dimer^10^, and S-trimer^20^ are being developed into vaccines. Besides, we demonstrated the potency of SARS-CoV-2 RBD in triggering neutralizing antibodies in several animal species such as mice and equines in a previous study, and the equine F(ab’)_2_ antibody showed a high-affinity and neutralizing capacity to SARS-CoV-2^21^.

Here, we reported a natural RBD-homodimer as a novel COVID-19 vaccine candidate. The RBD-homodimer was expressed in CHO cells with a high yield, and was linked covalently by an interdomain disulfide bond at S protein position 538 amino acid. The humoral and cellular responses were characterized in both rodents and nonhuman primates. The efficacy of the RBD vaccine was evaluated in mouse models and rhesus macaque model, which showed excellent protection from SARS-CoV-2 infection. Also, the antibodies collected from these vaccinated animals could bind to other RBD variants and neutralize the SARS-CoV-2 variants. These data highlighted that the RBD-homodimer reported in this study is an effective and high-yield vaccine candidate for preventing SARS-CoV-2 infection.

## Results

### Rational design, construction, purification, and characterization of RBD-homodimer

**Fig. 1:**
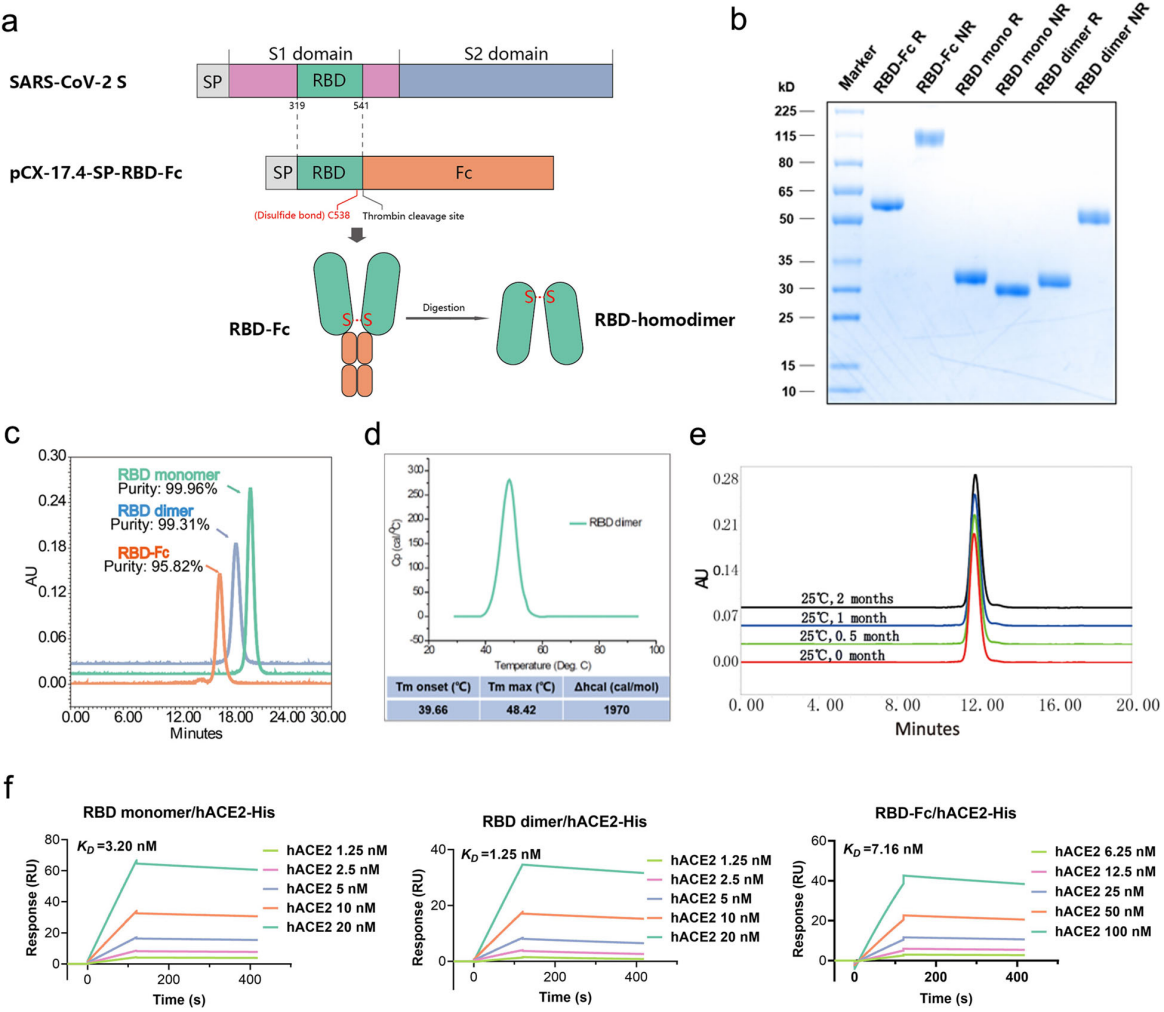
Rational design, purification, and characterization of RBD-homodimer. **a** Schematic diagram of the full-length SARS-CoV-2 spike protein (S) and RBD protein (319–541 amino acid). Structural elements include cleavable signal sequence (SP, gray), S1 (pink), and S2 (blue), receptor binding domain (RBD, green), thrombin cleavage site, Fc (orange), disulfide bond at S protein position 538 amino acid between RBD monomers (red). **b** Analysis of the reduced and non-reduced RBD proteins and RBD protein precursors (RBD-Fc) through SDS-PAGE and Coomassie brilliant blue staining. RBD was acquired from thrombin digestion on RBD-Fc and removing Fc fragments. R reduced form, NR non-reduced form. **c** Analysis of RBD monomer, RBD dimer, and RBD-Fc proteins by size exclusion chromatography (SEC), and the corresponding purities were marked in the graph. **d** Thermostability of RBD dimer proteins calculated by differential scanning calorimetry (DSC). **e** The long-term stability of RBD dimer at 25 °C was detected by SEC. **f** The binding affinity of RBD monomer, homodimer, and RBD-Fc to hACE2-His. The corresponding *K*_D_*s* were calculated using the Biacore T200 Evaluation 3.0 (software) with “1:1 binding” model as the curve fitting method.

Through the analysis of S protein of SARS-CoV-2, a cysteine was identified at the C-terminus of RBD, which might play a key role in the formation of stable dimers. With our previous experience of Fc fusion protein, the RBD and Fc fusion protein approach was taken to bring the C-terminal cysteines of the RBD protein closer, which increased the possibility of two RBD molecules forming disulfide bonds. According to this design, the SARS-CoV-2 RBD gene (319–541 amino acid) of the S protein, fused with the Fc gene of human IgG1, was constructed into the expression plasmid to obtain pCX-17.4-SP-RBD-Fc, which produced RBD dimer with the Fc tag. The Fc fragment was then removed by introducing a thrombin cleavage site to obtain stable RBD dimer (Fig. 1a).

Monoclonal CHO cell line (CHOK1SV GS-KO) with high-yield RBD was obtained by pressure screening after transiently transfecting the constructed plasmid into the CHO cells. The transfected cells were cultured with serum-free and protein-free medium, and RBD-Fc proteins were collected after filtered with depth filter, purified through protein A agarose affinity chromatography, and thrombin digestion to cut the Fc tag. The RBD protein was further purified by protein A agarose affinity chromatography to remove Fc fragments. Along with these processes, three components including RBD-Fc, RBD monomer, and RBD dimer were collected for characterization. Their sizes, purities, thermostabilities, and affinities to hACE2 were analyzed by sodium dodecyl sulfate-polyacrylamide gel electrophoresis (SDS-PAGE), high performance liquid chromatography-size exclusion chromatography (HPLC-SEC), differential scanning calorimetry (DSC), and surface plasmon resonance (SPR), respectively.

Through SDS-PAGE analysis, we found that the size of the non-reduced RBD dimer was about 50 kDa, which was about twice of the reduced monomer (Fig. 1b). The data together demonstrated that there are disulfide bonds between two RBD monomers, which is consistent with the previous reports on RBD structure^17^. After substituting the cysteine at position 538 with tryptophan, RBD could not form a dimer (Supplementary Fig. S1). These results indicated that the RBD protein formed a homodimer through the disulfide bond of inter Cys538-Cys538 residues. HPLC-SEC showed that the molecular weight of the three proteins was consistent with the theoretical value, and the purity of RBD-Fc, RBD monomer, and RBD dimer were 95.82%, 99.96%, and 99.31%, respectively (Fig. 1c), implying a high purity of RBD dimer to meet the medicinal standard. Furthermore, the thermal stability of the RBD dimer was determined by differential scanning calorimetry (DSC), and the thermal onset transition temperature was 39.66 °C and the *T*_max_ was 48.42 °C (Fig. 1d). The stability test for RBD dimer was conducted at 25 °C, and the results showed good stability of RBD dimer at 25 °C at least for 8 weeks (Fig. 1e). The affinity (*K*_D_) of RBD dimer to hACE2 was 1.25 nM, which is slightly better than that of the monomer RBD (3.20 nM) and RBD-Fc (7.16 nM) (Fig. 1f).

In summary, the RBD-homodimer showed high purity, high affinity to hACE2, and good stability, which are essential for vaccine development.

### The RBD-homodimer vaccine protects Ad5-hACE2-transduced BALB/c mice from SARS-CoV-2 infection

**Fig. 2:**
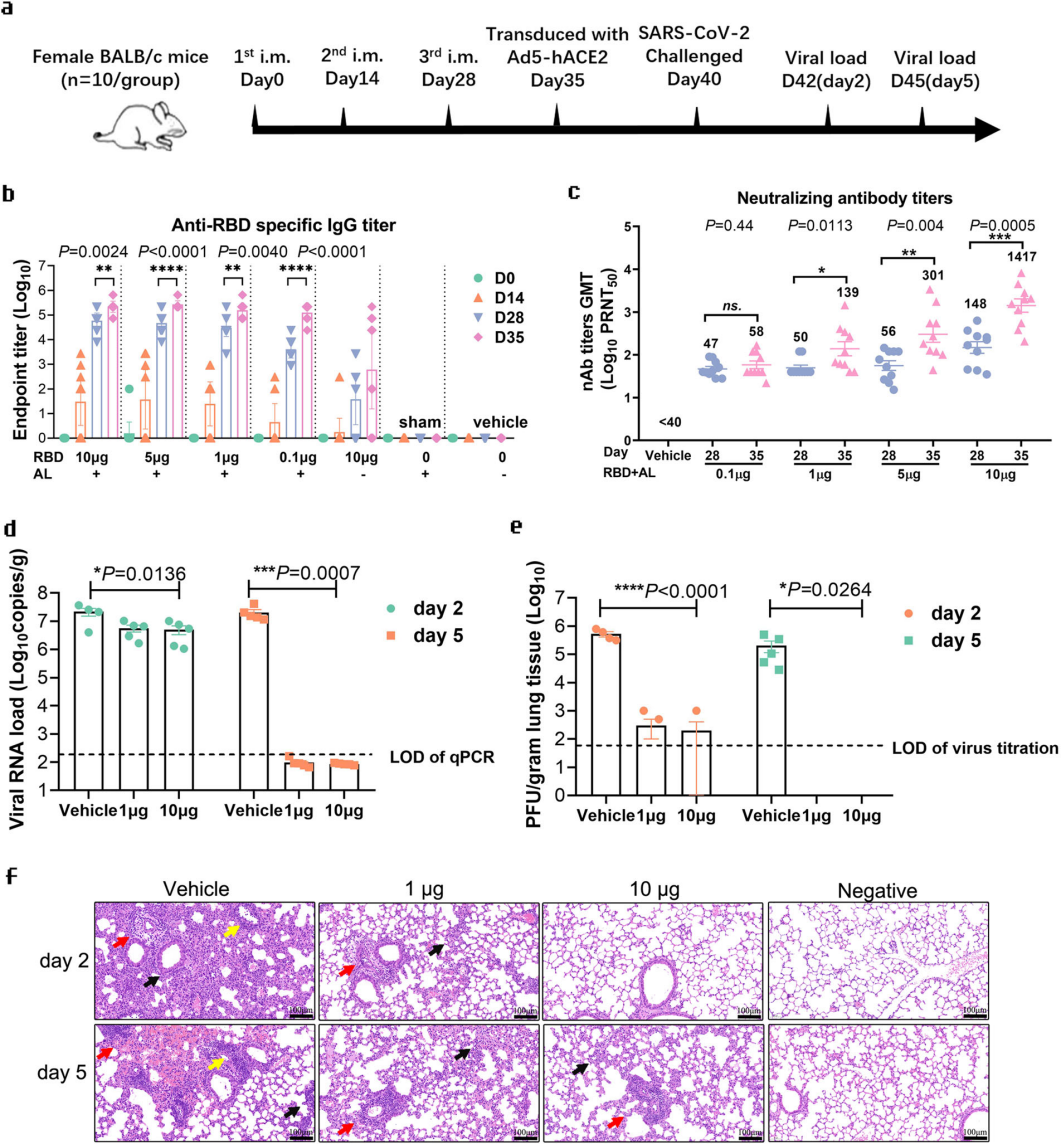
The RBD-homodimer vaccine protected Ad5-hACE2-transducted BALB/c mice from SARS-CoV-2 infection. **a** The experiment scheme. BALB/c mice were vaccinated at Day 0, Day 14, and Day 28. Mice of the 1 µg RBD-AL, 10 µg RBD-AL or AL only group were first transduced with Ad5-hACE2 (2.5 × 10^8^ PFU/mouse), then infected with 1.0 × 10^5^ PFU/mouse SARS-CoV-2 5 days later. At Day 2 or 5 of post-challenge, half of the mice from each group were euthanized for lung anatomy. AL aluminum adjuvant. **b** Bars indicated the geometric mean titer (GMT) of anti-SARS-CoV-2 RBD-specific IgG titers. Sera collected after the first immunization at different time points were indicated by different colors. The *x* axis showed the RBD dose and whether AL was added. **c** Sera collected from 0.1, 1, 5, and 10 µg RBD group at Day 28 and Day 35 were examined by PRNT, and the GMTs calculated from PRNT_50_ were presented. **d** Total viral genome copies in lung from each mouse post challenge were detected by RT-qPCR with a pair of primers targeting S gene under standard curve method. The limit of qPCR detection was 235 copies per reaction. The data were presented as means ± SEM. **e** Viral titer in lung from each mouse after SARS-CoV-2 challenge was detected by plaque formation on Vero E6 cells after a four-day infection, the limit of detection was 67 PFU/mL. **f** Lung tissues from mice dissected at Day 2 and Day 5 of post-challenge were executed pulmonary pathological detection by H&E staining. Representative pictures from Vehicle, 1 µg RBD, 10 µg RBD and blank group at Day 2 and Day 5 were displayed. Scale bars, 100 µm (20×). Arrow points the area appearing alveolar wall thickening, inflammatory cell infiltration, local bleeding, or other. *P < 0.05, **P < 0.01, ***P < 0.001, ****P < 0.0001.

To examine the immunogenicity of RBD dimers in vivo, RBD dimers formulated with or without Aluminum hydroxide adjuvant (AL) were used to vaccinate BALB/c mice. Animals were vaccinated three-shot regimens with 2-week intervals (Fig. 2a). The data in Fig. 2b showed that RBD dimer could elicit specific antibodies up to ~10^5^ of an endpoint titer after the third shot of 0.1, 1, 5, and 10 µg RBD protein with AL per mouse. The sera were also taken for measuring neutralizing antibody by plaque reduction neutralization test (PRNT). As shown in Fig. 2c, the geometric titer (GMT) of PRNT_50_ of the vehicle group was below 40, while the GMTs in 0.1, 1, 5, and 10 µg RBD group after the third vaccination (day 35) increased in a dose-dependent manner, and were higher than those after the second vaccination (day 28).

BALB/c mice transduced with hACE2 by recombinant Ad5-hACE2 were employed in this study to preliminarily evaluate the in vivo efficacy of the RBD dimer vaccine^22^. Mice were divided into three groups with ten mice in each: vehicle (AL only), 1 µg RBD, and 10 µg RBD group both with AL. Seven days after the third vaccination, the mice from each group were intranasally infected with 2.5 × 10^8^ plaque forming unit (PFU) Ad5-hACE2. On Day 5 after Ad5-hACE2 inoculation, all mice were intranasally challenged with 1.0 × 10^5^ PFU SARS-CoV-2. Mice were sacrificed at Day 2 and 5 of post-infection, and the lung tissues were harvested for viral detection and histopathology analysis.

As shown in Fig. 2d, total viral genome copies in lung tissue from the vehicle group was about 2.0 × 10^7^ copies/gram at 2 d.p.i., while the total viral genome copies in the 1 µg RBD and 10 µg RBD groups reduced to 5.7 × 10^5^ and 5.1 × 10^5^ copies/gram, about 1/40 of the vehicle group. At 5 d.p.i., total viral genome copies in lung tissue in vaccination groups went below the qPCR limit of detection (235 copies/reaction), while the viral load in the vehicle group still maintained at 2.0 × 10^7^ copies/gram. To measure the infectious particles from the supernatant of the homogenized lung tissue, plaque assay was performed to detect the live virus. The results showed the infectious viral titer was 5.0 × 10^5^ PFU/gram in the vehicle group at 2 d.p.i., while only a few infectious particles were detected in two mice in the 1 µg RBD group or one mouse in the 10 µg RBD group (Fig. 2e). At 5 d.p.i., the viral titer in the vehicle group was about 2.0 × 10^5^ PFU/gram. In contrast, no infectious particle was detected in the 1 µg RBD or 10 µg RBD group.

The lungs of the mice were also used for histopathological analysis, which revealed severe to moderate pulmonary pathological symptoms in vehicle group at 2 d.p.i. and 5 d.p.i., such as large area of alveolar wall thickening, local bleeding, and red blood cells leakage in the alveolar wall; large infiltration of inflammatory cells in the alveolar wall, bronchus and blood vessel, large vascular congestion were also observed (Fig. 2f). In contrast, only mild pulmonary pathological symptoms were observed in 1 µg RBD and 10 µg RBD group at 2 d.p.i. and 5 d.p.i.; there were bare infiltration of inflammatory cells in the alveolar wall, no vascular congestion was observed, and pulmonary alveolar was normal. Meanwhile, none of the pathological symptoms was observed in the lungs from blank mice at Day 2 or 5. Overall, the pathological symptoms caused by SARS-CoV-2 infection were greatly relieved by RBD dimer vaccination.

To examine the cellular response, spleens were harvested from RBD protein-immunized or adjuvant-immunized mice. The spleen cells were stimulated with RBD protein in vitro and the expressions of IL-4, IFN-γ, and IL-2 was checked. The data showed the fold changes of spot number in spleen cells from RBD-immunized mice secreting IL-4, IFN-γ, and IL-2, respectively, when compared to those from the adjuvant-immunized mice (Supplementary Fig. S2a).

Collectively, our data demonstrated that the RBD-homodimer vaccine showed great immunogenicity to elicit neutralization antibodies in mice, and the antibodies conferred protection from SARS-CoV-2 infection in mice.

### The RBD-homodimer vaccine protects hACE2-transgenic C57BL/6 mice from SARS-CoV-2 infection

**Fig. 3:**
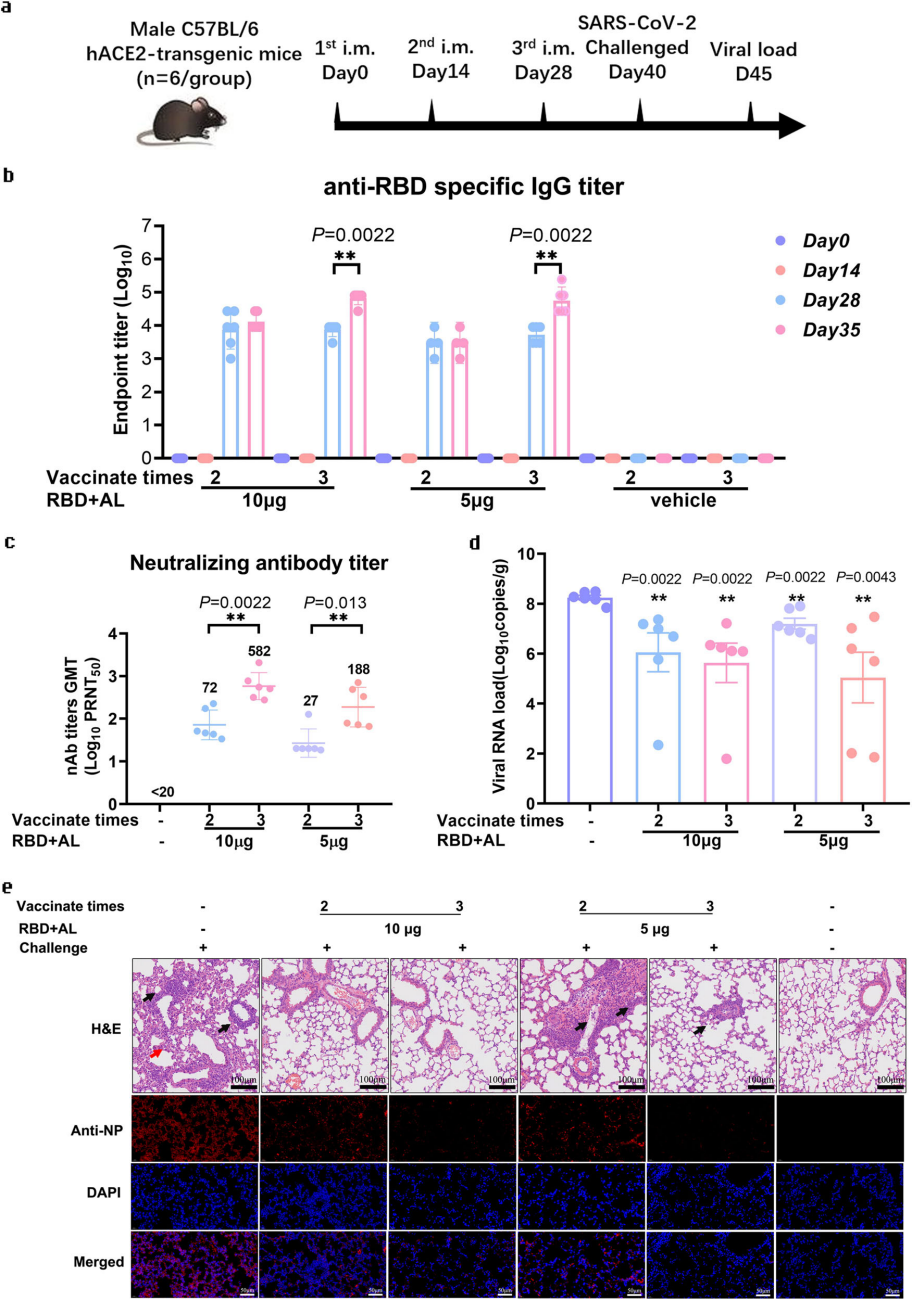
The RBD-homodimer vaccine protected hACE2-transgenic C57BL/6 mice from the infection of SARS-CoV-2. **a** The experiment scheme. Male C57BL/6 mice were vaccinated at Day 0 and Day 14, or Day 0, Day 14, and Day 28. Mice of the 5 µg RBD-AL adjuvant, 10 µg RBD-AL or AL only group were infected with 1.0 × 10^5^ PFU/mouse SARS-CoV-2 at Day 40. At 5 d.p.i., all the mice from each group were euthanized for lung anatomy. **b** Bars indicated the GMT of anti-SARS-CoV-2 RBD-specific IgG titers. Sera collected after each vaccination were detected by ELISA. The *x* axis showed the RBD dose and vaccinated times. **c** Sera collected from 5 and 10 µg RBD group after twice or three-time vaccination were examined by PRNT, and the GMTs calculated from PRNT_50_ were presented. **d** Total viral genome copies in lung from each mouse post challenge were detected by RT-qPCR with a pair of primers targeting S gene under standard curve method. The limit of qPCR detection was 235 copies per reaction. The data were presented as means ± SEM. **e** Lung tissues from mice dissected at 5 d.p.i. were executed pulmonary pathological detection by H&E staining (upper). Representative pictures from each group were displayed. Scale bar, 100 µm (20×). Arrow points the area appearing alveolar wall thickening, inflammatory cell infiltration, local bleeding, or other. Immunofluorescence analysis of viral antigens in lungs after challenge (lower). Sections were stained with antibodies targeting SARS-CoV-2 NP. DAPI was used to stain the nucleus. Scalar bars, 50 µm (40×). **P < 0.01.

To further evaluate the RBD-homodimer vaccine candidate in vivo, we employed another model, namely hACE2-transgenic C57BL/6 mice, to inspect more information. We routinely vaccinated mice (*n* = 6) with 5 or 10 µg RBD protein with AL as adjuvants. Mice were vaccinated two or three-shot regimen with 2-week intervals and challenged at Day 40 after the first vaccination (Fig. 3a). As shown in Fig. 3b, specific antibodies to RBD were effectively stimulated after the second vaccination (day 28), and the titers in 5 and 10 µg RBD group vaccinated two or three times reached to 10^5^–10^6^. Corresponding to that, the neutralizing titers in 5 and 10 µg RBD group after three-time vaccination were evaluated when compared with that after twice vaccination, and the GMT of PRNT_50_ in 5 and 10 µg RBD group vaccinated with three times were 188 and 582, respectively (Fig. 3c).

After intranasally challenging with 1.0 × 10^5^ PFU SARS-CoV-2, lung tissues were collected at 5 d.p.i. for both viral detection and histopathology analysis. Viral copies in all four vaccinated groups were significantly reduced (Fig. 3d). Viral copies in vehicle group reached 1.0 × 10^8^ per gram lung tissue, and those in 10 µg RBD group vaccinated (no matter twice or three times) were reduced by 2 logs, and that in 5 µg RBD group also decreased by 1 or 2 logs. The histopathological examination revealed severe to moderate pulmonary pathological symptoms in vehicle group when compared with blank group, e.g., large area of alveolar wall thickening, large infiltration of inflammatory cells in the alveolar wall, and large vascular congestion (Fig. 3e). At the same time, pulmonary pathological symptoms were obviously relieved in 10 µg RBD group vaccinated twice or three times and in 5 µg RBD group vaccinated three times. To confirm the infection of SARS-CoV-2 in lung, the lung sections were subjected to viral nucleoprotein (NP) examination by immunofluorescence. As shown in Fig. 3e, SARS-CoV-2 NP was abundantly expressed in lungs from the vehicle group, indicating that the SARS-CoV-2 infection model was successfully established. In comparison, only a small number of SARS-CoV-2 NP-positive cells were found in the lungs from 5 and 10 µg RBD group. In summary, our data in hACE2-transgenic C57BL/6 mice also proved that the RBD-homodimer could protect from SARS-CoV-2 infection in vivo, and implied that it is beneficial to choose a three-shot regimen.

### The RBD-homodimer vaccine protects rhesus macaques from SARS-CoV-2 infection

**Fig. 4:**
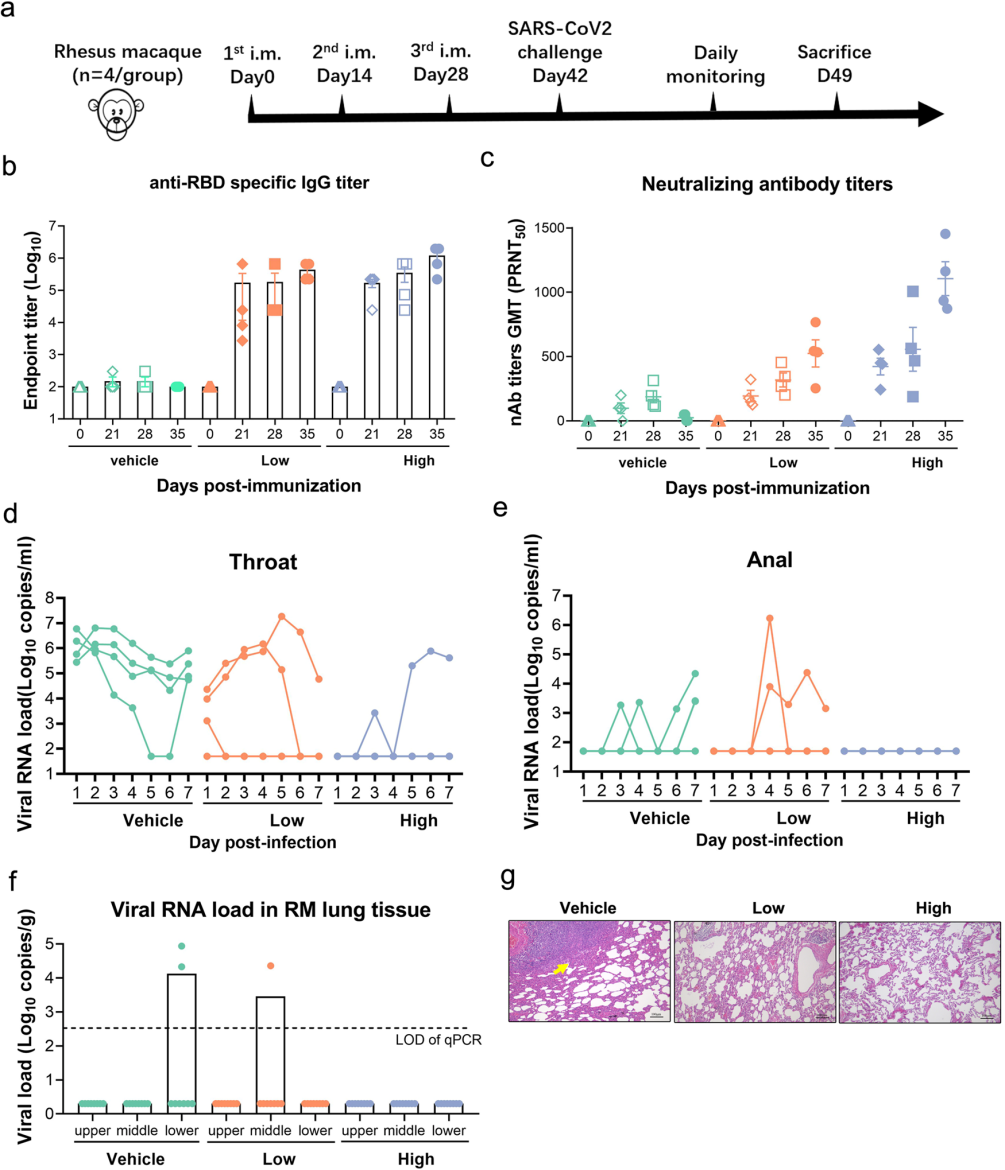
The RBD-homodimer vaccination protected RMs from SARS-CoV-2 challenge. **a** The experiment scheme. Four RMs were immunized via i.m. route with 25 µg or 50 µg of RBD vaccine at Day 0, 14, and 28, respectively. Four animals were injected with an equal volume of the adjuvant. At Day 42 of post-first shot, all the animals were challenged by SARS-CoV-2 (1.0 × 10^5^ TCID_50_). Viral loads in swabs were monitored to evaluate viral replication kinetics in RMs. The viral RNA was extracted to quantify viral copies. **b**, **c** Bars indicated the GMT of anti-RBD specific IgG titers (**b**) and neutralizing antibody titers (**c**) of live SARS-CoV-2. **d**, **e** RNA copies of throat (**d**) and anal swabs (**e**) from each RM through the experiment course. **f** Viral RNA load in the upper, middle, and lower lung at the endpoint from vehicle, low-dose, and high-dose group. The data were presented as means ± SEM. **g** Pulmonary pathology detected by H&E staining. Scalar bars, 100 µm (10×).

We initiated the study in 12 rhesus macaques (RMs) to evaluate the immunogenicity and efficacy of the RBD dimer vaccine. The RMs were divided into three groups with four animals (two males and two females) in each group: vehicle (AL only), low dose (25 µg RBD), and high dose (50 µg RBD). The RMs were vaccinated via intramuscular (i.m.) route at Day 0, 14, and 28. The RMs were bled weekly to monitor the antibody level. At Day 42 of post primary immunization, all the RMs were challenged by SARS-CoV-2 with dose 1.0 × 10^5^ TCID_50_ each by the intratracheal route (Fig. 4a).

The RBD-specific antibody in sera taken from the RMs was detected by Enzyme-linked immunosorbent assay (ELISA). Figure 4b showed the specific antibodies titers after each vaccine immunization with 25 or 50 µg RBD. The neutralizing antibody in sera taken from the RMs was detected by PRNT. As shown in Fig. 4c, the GMT of PRNT_50_ of the 25 µg and 50 µg RBD group after the third (day 35) vaccination was 485 and 1083, respectively. Upon infection, no obvious clinical signs were observed during the 7-day study course in all animals, and the RMs did not show any changes in the body weight (Supplementary Fig. S3a) or body temperature (Supplementary Fig. S3b). The neutralizing antibody titers of RM sera post-challenge were attached as Supplementary Fig. S4.

In the throat swab samples, the peak viral RNA loads in three out of four animals from the vehicle group in oropharyngeal swab ranged from 1.3 × 10^5^ to 6.4 × 10^6^ copies/mL, while one animal showed the decreasing RNA level from day 1 to day 6 post infection with the amount of 1.9 × 10^6^ copies/mL to an undetectable level and the amount of RNA reached to 7.7 × 10^4^ copies/mL at Day 7 (Fig. 4d). In contrast, the RNA was undetectable in three out of four animals from the high-dose group and one animal showed positive on day 3, 5–7 of post infection with 7.7 × 10^5^ copies/mL at Day 6. In the low-dose group, all the animals showed at least 12 times lower viral RNA at 1 d.p.i., and the virus was cleared at Day 6 and 7 in three animals. In the anal swab samples, viral RNA was detected in the vehicle group animals at discrete time points, while viral RNA was detected in two RMs from the low-dose group. No viral RNA was detected in RMs in the high-dose group (Fig. 4e). Similar to the results from the mice, the fold changes of the spot number of IL-4-secreted, IFN-γ-secreted, and IL-2-secreted PBMCs from RBD-immunized RMs were significantly increased compared to the control group (Supplementary Fig. S2b).

In order to further understand the distribution of the virus in different tissues of the upper respiratory tract and lungs, the RMs were sacrificed and tissues from different lobes of the lung in the control group, the low dose group, and the high dose group were collected at Day 7 of post-challenge. As shown in Fig. 4f, viral RNA was detected in the lower lobe of two RMs in the vehicle group, and could be detected in the middle lobe of one RM in the low dose group, while no viral RNA could be detected in the upper, middle, or lower lobe of the animals in the high dose group. The lobes were fixed and sectioned for histopathological analysis. Three RMs from control groups displayed moderate interstitial pneumonia, characterized by thickened alveolar septa accompanied by infiltration of inflammatory cells in a large area of the lung tissues. In contrast, the severity of lesions in the lung tissue in the RMs vaccinated with the RBD was significantly reduced (Fig. 4g). Peripheral blood mononuclear cells (PBMCs) from RBD protein-immunized or adjuvant-immunized RMs were also harvested to test the production of cytokines after stimulation with RBD protein in vitro. Taken together, the neutralizing antibody stimulated by RBD-homodimer vaccination conferred protection against SARS-CoV-2 infection to immunized RMs.

### Cross-neutralization on SARS-CoV-2 strains by RM sera elicited by RBD homodimer

**Fig. 5:**
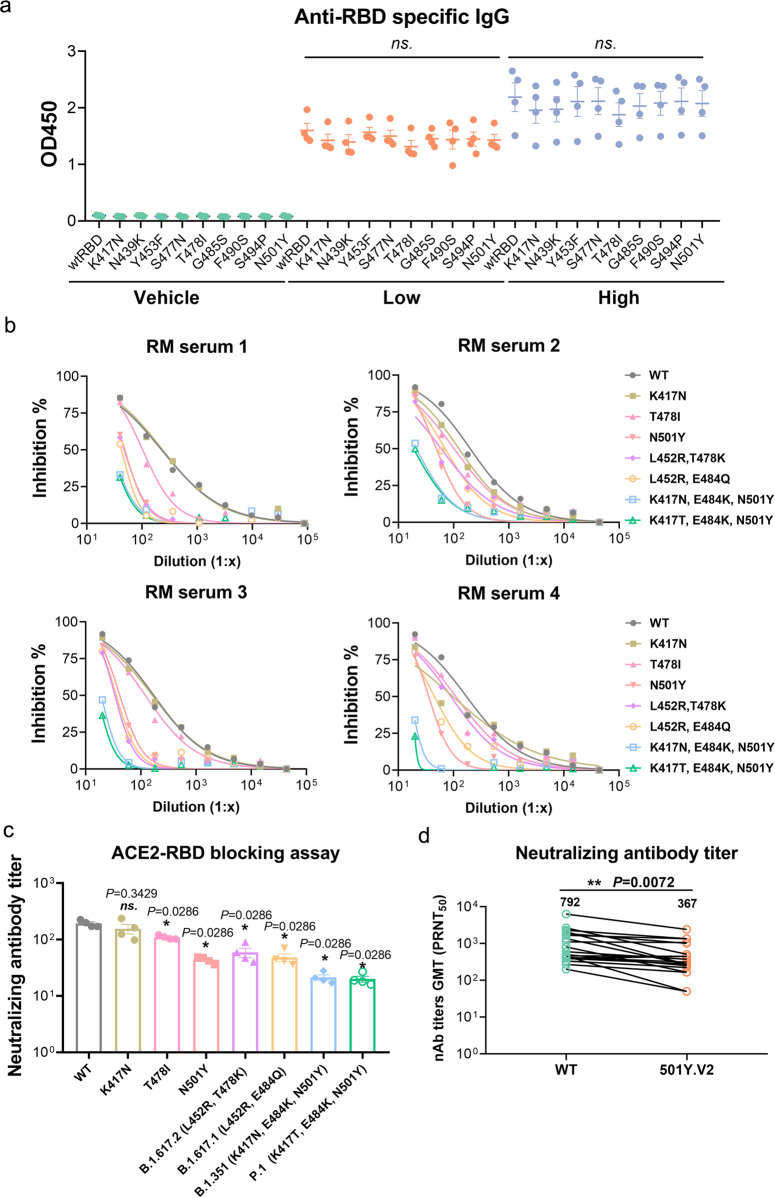
The cross-neutralization capacity of RM sera elicited by RBD-homodimer on SARS-CoV-2 variants. **a** The OD450 of four RM sera (1:400 diluted) elicited by WT RBD dimer was detected on nine eukaryotic RBD variants and one WT RBD with conventional ELISA. **b** Sera adopted from four vaccinated RMs were detected by hACE2-RBD blocking assays. The mutants were presented, and the inhibition% was equal to blocking rate. **c** The neutralizing antibody titers calculated from **b** were displayed (unpaired *t*-test with WT). **d** The neutralizing antibody titer of twenty RM sera vaccinated with RBD dimer against both WT and 501Y.V2 were detected by PRNT (paired *t*-test). ns, *P* > 0.05, *P < 0.05, **P < 0.01.

Currently, the SARS-CoV-2 variants were reported worldwide with the potential to enhance viral transmission and virulence. To further explore the effectiveness of the RBD dimer vaccine on SARS-CoV-2 variants, we tested the binding and neutralization capacity of the antibodies elicited by the RBD dimer to the nine RBD variants including mutations at K417N, N439K, Y453F, S477N, T478I, G485S, F490S, S494P, and N501Y (purchased from Sino Biological Inc.). As shown in Fig. 5a, sera collected from RMs vaccinated with RBD dimer (wild type, WT) could bind all of these nine mutant RBD proteins, while their binding capacities to several mutants (K417N/N439K/T478I) slightly decreased.

To further explore the cross-protection of the RBD homodimer from the infection of SARS-CoV-2 strains, we employed hACE2-RBD blocking assay on different RBD mutants by four RM sera vaccinated by RBD homodimer. In Fig. 5b, the inhibition of four sera on the binding of RBDs to ACE2 were presented and their neutralizing antibody titers were summarized in Fig. 5c. As a consequence, the mutation at N501Y in RBD reduced the neutralization of sera the most among the three mutations K417N, T478I, and N501Y, while the mutation at K417N reduced that the least. Moreover, mutations as L452R&T478K (Delta), L452R&E484Q (Kappa), K417N&E484K&N501Y (Beta, 501Y.V2), and K417T&E484K&N501Y (Gamma, 501Y.V3), which represent SARS-CoV-2 lineage as B.1.617.2, B.1.617.1, B.1.351, and P.1, respectively, reduced the neutralization to varying degrees.

Since the key mutations K417N/T, E484K, and N501Y in RBD that may affect immune response are all included in the South Africa isolate, 501Y.V2^23^, we tested the sera elicited by the RBD dimer through a set of neutralization experiments on both WT and South Africa isolates. Our results showed that sera elicited by WT RBD could potently neutralize both isolates, while with one-fold decrease in PRNT_50_ on the South Africa isolate (Fig. 5d). To some extent, these data implied that vaccine RBD dimer may provide cross-protection from infection by SARS-CoV-2 variants, while showed a decrease in neutralization in vitro.

## Discussion

The COVID-19 pandemic is still going on worldwide. Despite that numerous vaccine candidates are under development or granted for emergency use, we are still facing a huge shortage in distributing the vaccine globally, especially in developing countries. The RBD dimer reported in this study, which shows robust immunogenicity and efficacy both in mice and non-human primates, is a potent COVID-19 vaccine candidate and worth further development. Different from other protein subunit vaccines, the RBD developed here is a natural homodimer without introducing exogenous amino acids, and the dimer is considered to be strong immunogen in eliciting high neutralizing antibodies in vivo in our study (Supplementary Fig. S5), which exhibits superiority as a vaccine and has been verified by previous study. Recently, Dai et al. has demonstrated that an artificial tandem coronavirus RBD dimer was more efficient in triggering antibody response than monomer in rodents^10^. Coincidently, Jiang et al. reported that SARS-CoV-2 RBD-Fc dimer elicits high titer of neutralizing antibodies in vivo, and protects hACE2-transgenic mice from SARS-CoV-2 challenge^24^. On the basis of these, our research confirmed that a dimeric RBD is a better choice to further develop as a vaccine candidate.

As one of the hottest topics for COVID-19 vaccine, whether the circulating SARS-CoV-2 variants can escape neutralizing antibodies elicited by vaccines, or whether approved vaccines can confer protection in vivo is still a mystery. Since SARS-CoV-2 is an RNA virus with low fidelity in RNA-dependent RNA polymerase, the high transmissibility leads to its rapid spread across different species and continents and evolved into seven new lineages including B.1.1.7 (Alpha), B.1.351 (Beta), P.1 (Gamma, 501Y.V2), B.1.617 (Delta), B.1.429 (Epsilon), B.1.525 (Eta), and B.1.617.1 (Kappa)^23,25,26^. Key mutations in S protein usually affect the transmissibility, virulence, or immune response. For instance, the D614G in RBD was reported to increase the infectivity and transmission of SARS-CoV-2^27–30^, while has less impact on the vaccine effectiveness^31^. Different from D614G, mutations K417N/T, E484K, and N501Y especially the mutation at position 484 in 501Y.V2 have the capacity to evade immune response^25,32^. Such mutations within the binding epitopes of antibodies might affect immunogenicity of RBD and render antibodies ineffective^33^. For now, the neutralizing antibodies elicited by Pfizer and Moderna vaccines all showed less effectiveness on variant 501Y.V2 compared to the initial strain^23,34–38^, and vaccines or convalescent sera exhibited reduced neutralization against SARS-CoV-2 B.1.617^39–41^. In addition, the data from RBD vaccine and inactivated vaccine showed the preserved neutralizing capacity with a slightly reduction against 501Y.V2 authentic virus when compared with the original strain^34^. Based on our data, the RBD vaccine candidate developed in this study showed a similar decrease tendency in neutralization against several strains, urging for new strategies to confront the rigorous epidemic situation on the basis of present vaccines.

Different vaccines work differently, and their capacity in triggering humoral and cellular immune responses varied. For instance, the adenovirus-vector vaccine and nucleic acid vaccine are more effective in stimulating the cellular immune response^42^, while the protein subunit vaccine and inactivated-virus vaccine are on the contrary. Among the approved vaccines, adenovirus-vector vaccine and mRNA vaccines need one or two-shot regimen^8,43–45^, while inactivated-virus vaccine and protein subunit vaccine are in favor of two-shot or three-shot regimen^4,11,46–48^. The basic principle is to ensure that the titers of neutralizing antibodies is high enough to reduce the incidences of infection, and severe illness as well as to resist variant strains^49^. The results from our study suggested that a three-shot regimen is more effective, and can ensure a high neutralizing antibody titer in vivo thereby resisting the SARS-CoV-2 variants.

In summary, the RBD-homodimer vaccine candidate reported here can induce both humoral and cellular response in vivo and prevent SARS-CoV-2 infection in both rodents and non-human primates. Furthermore, the production of this RBD vaccine candidate can be performed in large scale, which will enhance the vaccine supply to the world and warrants future clinical trials.

## Materials and methods

### Cells lines and viruses

Vero E6 cells were maintained in Dulbecco’s modified Eagle’s medium (DMEM, Gibco, NY, USA) supplemented with 10% fetal bovine serum (FBS, Gibco, NY, USA) at 37 °C with 5% CO_2_. The CHOK1SV GS-KO host cells (Lonza, Switzerland) were thawed and cultured in a chemical-defined serum-free CD CHO medium. ExpiCHO-S cells were cultured in ExpiCHO Expression Medium (Gibco, NY, USA).

Live SARS-CoV-2 (nCoV-2019BetaCoV/Wuhan/WIV04/2019) and South Africa variant (501Y.V2) were obtained from the National Virus Resource, Wuhan Institute of Virology, Chinese Academy of Sciences, and handled in a BSL-3 laboratory. Virus titrations were performed by endpoint titration in Vero E6 cells.

### Plasmid construction, protein expression, and purification

The sequence of the SARS-CoV-2 RBD gene (amino acids R319-F541, Genbank: QHR63260.2) was codon-optimized for mammalian cell expression and synthesized by GenScript Co., Ltd., and then cloned into the expression plasmid pXC17.4 (LONZA) to obtain RBD-(Thrombin site)-Fc. Fc is in the form of WT human IgG1.

After the expression plasmid was transfected into CHO cells, high-expressing candidate clones were obtained by pressure screening. Cell line development and large-scale production were generated by Wuhan YZY Biopharma. Co. Ltd., China. After cultured in a 250 L fermentor, the RBD homodimer without an Fc tag was obtained from RBD-Fc by removing the Fc region through thrombin digestion. The RBD proteins were collected from the flow-through fraction, and affinity chromatography against Protein A was repeatedly conducted to completely remove the residual Fc regions. The final yield of RBD homodimer was about 1 g/L.

hACE2-Fc (amino residues 1–709, Genbank: Q9BYF1) was codon-optimized and cloned into expression plasmid pXC17.4 (LONZA) with an Fc tag at the C terminus. After mutating the cysteine at position 538 to tryptophan, RBD C538S was cloned into the expression plasmid pXC17.4 (LONZA) to obtain RBD C538S-(Thrombin site)-Fc. The plasmid was transiently transfected into ExpiCHO-S cells using an ExpiFectamine Transfection Kit. After 8-day culture, the culture supernatant was collected, and the soluble protein was sequentially purified by protein A-affinity chromatography and ion exchange chromatography. hACE2-His (Cat No: 10108-H08H) was expressed from HEK293T cells with poly-histidine tag at the C terminus (Sino Biological Inc., China).

### Reduced and non-reduced SDS-PAGE

The prepared RBD-Fc and RBD proteins were analyzed by SDS-PAGE with Coomassie Brilliant Blue staining. To verify the sizes and purities of these proteins, reducing and non-reducing SDS-PAGE were employed. The reduced and non-reduced samples were prepared by mixing 2 μg of the proteins with loading buffer, adding 2-β-mercaptoethanol or not, and the mixtures were boiled in water for 10 min. The samples were concentrated via 4–20% SDS-PAGE (GenScript, China). Finally, the SDS-PAGE gels were stained with Coomassie Brilliant Blue. After decolorization, images of the gels were captured with a ChemiDoc MP Imaging system (Bio-Rad, CA, USA).

### SPR

The SPR assays were carried out using BIAcore T200 (GE Healthcare). The RBD monomer, dimer, and RBD-Fc were immobilized on CM5 chips (GE Healthcare), respectively. hACE2-His was diluted as 20, 10, 5, 2.5, and 1.25 nM to bind to RBD dimer and RBD monomer, and diluted as 100, 50, 25, 12.5, and 6.25 nM to bind to RBD-Fc. The interaction was determined with the flow rate of 30 μL/min, and the association time is 120 s and the dissociation time is 300 s. The chip was regenerated with pH 1.5 Glycine solution. The values of respective *K*_D_ were calculated using the Biacore T200 Evaluation 3.0 (software) with “1:1 binding” as the curve fitting method.

### Vaccine formulation and stability evaluation

The purified RBD protein was formulated in 20 mM Citrate, 0.85% NaCl, 0.04% Tween-80, pH 5.5. Samples and corresponding buffers were loaded in MicroCal VP-Capillary (GE, US) with a volume of 400 μL. The sample and control buffer were heated at the same time, from 25 to 100 °C, at a rate of 2 °C/min. After the detection was completed, the control buffer background was subtracted and the baseline is corrected to obtain the DSC spectrum.

### ELISA

96-well ELISA plates were coated with RBD protein of 2 μg/mL at a volume of 100 μL/well overnight at 4 °C. The plates were washed three times with PBST (PBS with 0.1% Tween-20) and then blocked with 3% BSA at 37 °C for 1 h. Gradient-diluted sera were added to each well, and the plates were incubated at room temperature for 1 h. Then the plates were washed three times with PBST, and incubated with HRP-conjugated secondary antibodies at room temperature for 1 h. After the plates were washed three times as described above and added 100 μL/well of TMB solution (BD, USA) to all the wells. After 0.5 h at room temperature, the plates were added 100 μL of 2 M HCl to all the wells and the absorbance at 450 nm was measured with LUX 3020 microplate reader (Thermo, USA), and samples with values greater than twice those of the controls were considered positive.

### Enzyme-linked immunospot assay (ELISPOT)

Mice splenocytes (5.0 × 10^5^/well) and rhesus macaques PMBCs were added to the IL-2/IL-4/IFN-γ Ab pre-coated plate kit (MabTech, USA). Then, RBD proteins were added to the wells. Phytohemagglutinin (PHA) was added as a positive control. Cells without stimulation were employed as a negative control. After 36 h of incubation, the cells were removed, and the plates were processed in turn with biotinylated detection antibody. After the spots grow to a suitable size, the liquid was poured, the base of the plate was covered, and the front and back sides were washed with water to stop the color development process. The ELISPOT plate was then placed in the Mabtech IRISTM automatic plate reader, and the parameters for spot counting were adjusted, and statistical analysis was performed.

### PRNT

Vero E6 cells were seeded in a 24-well plate with 1.0 × 10^5^ cells/well overnight. The next day, sera from mice or rhesus macaque were serially diluted with DMEM and incubated with SARS-CoV-2 at 37 °C for 1 h, and DMEM without sera served as a negative control. Then, the mixture was added to the monolayer Vero E6 cells. After incubation at 37 °C for 1 h, the supernatants were completely removed, and the cells were washed with PBS and covered with 0.9% methylcellulose-2% FBS-DMEM. Four days later, the covering was removed, cells were fixed with 4% paraformaldehyde and stained with 1% crystal violet. The plaques were manually counted.

### Vaccination and challenge of mice

Female BALB/c mice aged 6–8 weeks (*n* = 10) or male hACE2-transgenic C57BL/6 mice (*n* = 6) were housed in specific pathogen-free animal care facilities. According to a homogeneous prime-boost-boost protocol, immunization was performed twice or three times by i.m. injection with two-week intervals. In detail, RBD proteins mixed with an equal volume of aluminum hydroxide adjuvant (Croda, UK) for priming or boosting. A total of 300 μL of the mixture was injected into each mouse. Blood samples were collected from the ophthalmic vein 7 or 14 days after each immunization. To comply with animal 3R principles, the spare mice would be used for internal research.

BALB/c mice were lightly anesthetized with isoflurane via airway, then 2.5 × 10^8^ PFU Ad5-hACE2 viruses in 50 μL DMEM were delivered via nasal cavity to the lung of mice to complete human ACE2 transduction. Five days later, Ad5-hACE2-transducted BALB/c mice or hACE2-transgenic C57BL/6 mice were intranasally challenged with SARS-CoV-2 (1.0 × 10^5^ PFU) after airway anesthesia. Thereafter, the body weight of each mouse was daily recorded from Day 0 to Day 5. At Day 2, half of the BALB/c mice were dissected after orbital blood sampling, and the left lung was taken for histopathology examination, and the right lung was taken for viral genome copy detection and virus titer detection. At Day 5, the other half of the BALB/c mice or all the C57BL/6 mice were treated the same way.

### Virus detection in mouse lung tissue

For detection of the virus in lung tissue, two methods were employed. One was to detect the viral genome copies via RT-qPCR, and the other was to detect the live virus by plaque formation assay.

In detail, the right lung was homogenized in 1 mL DMEM with a tissue grinding apparatus. Then 200 μL homogenized fluid was subjected to RNA isolation with a Qiagen RNeasy Mini kit, and the total RNA was eluted with 30 μL RNase-free water. cDNA was transcribed from 3 μL total RNA in 20 μL reaction system with PrimeScript^TM^ RT reagent Kit with gDNA Eraser (Takara). Viral copies were quantified from 1 μL cDNA template viral cDNA by a standard curve method on ABI 7500 (Takara TB Green® Premix Ex Taq™ II) with a pair of primers targeting S gene. The forward primer (5′-3′): CAATGGTTTAACAGGCACAGG; the reverse primer (5′-3′): CTCAAGTGTCTGTGGATCACG. The standard curve was set from eight points in 20 µL reaction system (2.35 × 10^9^ copies, 2.35 × 10^8^ copies, 2.35 × 10^7^ copies, 2.35 × 10^6^ copies, 2.35 × 10^5^ copies, 2.35 × 10^4^ copies, 2.35 × 10^3^ copies, 2.35 × 10^2^ copies). Copies of samples <2.35 × 10^2^ copies were defined as negative. The copies of positive samples were converted with the equation: (sample well-blank well) copies × 20 × 10/0.2 mL/g lung tissue in 1 mL, and presented in the figure.

Or, 50 μL homogenized fluid was directly taken for 10-fold gradient dilution (10^−1^, 10^−2^, 10^−3^, 10^−4^, 10^−5^, 10^−6^), and 150 μL diluted fluid were inoculated to infecting Vero E6 monolayer cells seeded in 24-well plate for 1 h. Then the supernatants were completely removed, and cells were washed once with PBS, and covered with 0.9% methylcellulose-2% FBS-DMEM. Four days later, the covering was removed, and cells were fixed with 4% paraformaldehyde and stained with 1% crystal violet. The plaques were manually counted and the virus titer was calculated as usual, no plaque at 10^−1^ was defined as negative, and the limit of the plaque experiments was about 67 PFU/mL.

### Vaccination and challenge of RMs

Twelve RMs (age 5–7 years old, weight 5–7 kg) were divided into three groups and inoculated with AL only, 25 µg RBD vaccine, or 50 µg RBD vaccine on Day 0, 14, and 28. The blood was bled on Day 0, 7, 14, 21, 28, and 35, and sera were collected for RBD-specific and neutralizing antibody test. The RMs were challenged on Day 42 after primary immunization with dose 1.0 × 10^5^ TCID_50_/monkey intratracheally. The RMs were observed daily, with detailed recording of clinical signs, symptoms, morbidity, and mortality, including the nature, onset, severity, and duration of all gross or visible changes. Swab samples of the oropharyngeal, nasal turbinate, and rectal regions were collected at 1–7 d.p.i. To confirm the pathogenesis and injury in the respiratory tract, all animals were sacrificed at 7 d.p.i. All six lung lobes were collected on the day of euthanization for various pathological, virological, and immunological analysis.

Viral RNA in the samples was quantified by one-step real-time quantitative RT-PCR. The swab and blood samples were used to extract viral RNA by using the QIAamp Viral RNA Mini Kit (Qiagen), according to the manufacturer’s instructions. Tissues were homogenized in DMEM (1:10, W/V), clarified by low-speed centrifugation at 4500×*g* for 30 min at 4 °C, and supernatant was immediately used for RNA extraction. RNA was eluted in 50 μL of elution buffer and used as the template for RT-qPCR. The primer pairs were used following our previous study, which is targeting S gene. Two microliters of RNA were used to verify the RNA quantity by HiScript® II One Step qRT-PCR SYBR® Green Kit (Vazyme Biotech Co., Ltd.) according to the manufacturer’s instructions. The amplification was performed as followed: 50 °C for 3 min, 95 °C for 30 s followed by 40 cycles consisting of 95 °C for 10 s, 60 °C for 30 s, and a default melting curve step in an ABI StepOne machine.

### Histopathology and immunofluorescence

Lung tissues after dissection were fixed in formalin. Tissue block after dehydration and paraffin embedding was sectioned as 4 mm each and then stained with hematoxylin and eosin (H&E). To detect virus antigen, sections were first blocked with BSA and then incubated with a rabbit polyclonal antibody to SARS-CoV-2 N protein (1:500, from Prof. Zhengli Shi at Wuhan Institute of virology), then incubated with fluorescent secondary antibodies, followed by incubation with DAPI (Sigma-Aldrich, D9542). Images were collected using a fluorescent microscopy (Pannoramic MIDI, 3D HISTECH).

### RBD-hACE2 blocking assay

The hACE2-Fc protein was diluted to 2.0 μg/mL and coated onto 96-well plates at a volume of 100 μL/well overnight at 4 °C. The plates were washed with 300 μL/well of 0.1% Tween 20-PBS for three times, and blocked with 200 μL/well of 1% BSA at 37 °C for 1 h. Then gradient-diluted mouse or RM sera were incubated with HRP-RBDs or RBD-His (Sino Biological Inc., Beijing, China) for half an hour, and incubated with the plate at 37 °C for 1 h. Then the plates were washed three times with PBST, and incubated with HRP-conjugated anti-His secondary antibodies (Proteintech, China) at room temperature for 1 h, or added 100 μL/well of TMB solution (BD, USA) to all the wells. After incubation at room temperature for 0.5 h, 100 μL of 2 M HCl were added to all the wells. The absorbance at 450 nm were measured with LUX 3020 microplate reader (Thermo, USA). The method of calculating the blocking rate (inhibition%) was: blocking rate = (1 – the value of OD450 nm detection of the test product/the value of OD450 nm detection of the negative control) × 100%, and the neutralizing antibody titers were calculated from inhibition%.

### Ethics statements

Non-human primate experiments were conducted within the animal biosafety level 4 (ABSL-4) facility and mouse experiments were performed in animal biosafety level 3 (ABSL-3) laboratory in the National Biosafety Laboratory (Wuhan), Chinese Academy of Sciences. All animal experiments were performed in strict accordance with the Regulations for the Administration of Affairs Concerning Experimental Animals in China, and the protocols were approved by the Laboratory Animal Care and Use Committee of Wuhan Institute of Virology, Chinese Academy of Sciences (Wuhan, China) (Ethics number: WIVA42202002).

### Data analysis

The data were processed using GraphPad Prism 8.0 software (CA, USA) and are presented as the means ± SEM based on at least three independent experiments. The statistical analysis was performed using unpaired or paired *t*-Test or one-way ANOVA. *P* values were defined as ns, *P* > 0.05, *P < 0.05, **P < 0.01, and ***P < 0.001.

## Supplementary information

Supplementary Information

## References

[1] Dai L, Gao GF (2021). Viral targets for vaccines against COVID-19. Nat. Rev. Immunol..

[2] Wouters OJ (2021). Challenges in ensuring global access to COVID-19 vaccines: production, affordability, allocation, and deployment. Lancet.

[3] Xia S (2020). Effect of an inactivated vaccine against SARS-CoV-2 on safety and immunogenicity outcomes: interim analysis of 2 randomized clinical trials. JAMA.

[4] Xia S (2021). Safety and immunogenicity of an inactivated SARS-CoV-2 vaccine, BBIBP-CorV: a randomised, double-blind, placebo-controlled, phase 1/2 trial. Lancet Infect. Dis..

[5] Wu Z (2021). Safety, tolerability, and immunogenicity of an inactivated SARS-CoV-2 vaccine (CoronaVac) in healthy adults aged 60 years and older: a randomised, double-blind, placebo-controlled, phase 1/2 clinical trial. Lancet Infect. Dis..

[6] Polack FP (2020). Safety and Efficacy of the BNT162b2 mRNA Covid-19 Vaccine. N. Engl. J. Med..

[7] Baden LR (2021). Efficacy and Safety of the mRNA-1273 SARS-CoV-2 Vaccine. N. Engl. J. Med..

[8] Ramasamy MN (2021). Safety and immunogenicity of ChAdOx1 nCoV-19 vaccine administered in a prime-boost regimen in young and old adults (COV002): a single-blind, randomised, controlled, phase 2/3 trial. Lancet.

[9] Zhu FC (2020). Safety, tolerability, and immunogenicity of a recombinant adenovirus type-5 vectored COVID-19 vaccine: a dose-escalation, open-label, non-randomised, first-in-human trial. Lancet.

[10] Dai L (2020). A universal design of betacoronavirus vaccines against COVID-19, MERS, and SARS. Cell.

[11] Keech C (2020). Phase 1-2 trial of a SARS-CoV-2 recombinant spike protein nanoparticle vaccine. N. Engl. J. Med..

[12] Zhou P, Shi ZL (2021). SARS-CoV-2 spillover events. Science.

[13] Zhou P (2020). A pneumonia outbreak associated with a new coronavirus of probable bat origin. Nature.

[14] Hu B, Guo H, Zhou P, Shi ZL (2021). Characteristics of SARS-CoV-2 and COVID-19. Nat. Rev. Microbiol..

[15] Jiang RD (2020). Pathogenesis of SARS-CoV-2 in transgenic mice expressing human angiotensin-converting enzyme 2. Cell.

[16] Shan C (2020). Infection with novel coronavirus (SARS-CoV-2) causes pneumonia in Rhesus macaques. Cell Res..

[17] Lan J (2020). Structure of the SARS-CoV-2 spike receptor-binding domain bound to the ACE2 receptor. Nature.

[18] Tai W (2020). Characterization of the receptor-binding domain (RBD) of 2019 novel coronavirus: implication for development of RBD protein as a viral attachment inhibitor and vaccine. Cell Mol. Immunol..

[19] Yang J (2020). A vaccine targeting the RBD of the S protein of SARS-CoV-2 induces protective immunity. Nature.

[20] Tian JH (2021). SARS-CoV-2 spike glycoprotein vaccine candidate NVX-CoV2373 immunogenicity in baboons and protection in mice. Nat. Commun..

[21] Pan X (2020). Immunoglobulin fragment F(ab’)2 against RBD potently neutralizes SARS-CoV-2 in vitro. Antivir. Res..

[22] Sun J (2020). Generation of a broadly useful model for COVID-19 pathogenesis, vaccination, and treatment. Cell.

[23] Garcia-Beltran WF (2021). Circulating SARS-CoV-2 variants escape neutralization by vaccine-induced humoral immunity. Cell.

[24] Liu Z (2020). RBD-Fc-based COVID-19 vaccine candidate induces highly potent SARS-CoV-2 neutralizing antibody response. Signal Transduct. Target Ther..

[25] Liu, Y. et al. The N501Y spike substitution enhances SARS-CoV-2 transmission. *bioRxiv*10.1101/2021.03.08.434499 (2021). 10.1038/s41586-021-04245-0 PMC8900207 34818667

[26] Duong D (2021). Alpha, Beta, Delta, Gamma: What’s important to know about SARS-CoV-2 variants of concern?. CMAJ.

[27] Gobeil SM (2021). D614G mutation alters SARS-CoV-2 spike conformation and enhances protease cleavage at the S1/S2 junction. Cell Rep..

[28] Zhang L (2020). SARS-CoV-2 spike-protein D614G mutation increases virion spike density and infectivity. Nat. Commun..

[29] Plante JA (2021). Spike mutation D614G alters SARS-CoV-2 fitness. Nature.

[30] Hou YJ (2020). SARS-CoV-2 D614G variant exhibits efficient replication ex vivo and transmission in vivo. Science.

[31] Yurkovetskiy L (2020). Structural and functional analysis of the D614G SARS-CoV-2 spike protein variant. Cell.

[32] Liu Z (2021). Identification of SARS-CoV-2 spike mutations that attenuate monoclonal and serum antibody neutralization. Cell Host Microbe.

[33] Li C (2021). The impact of receptor-binding domain natural mutations on antibody recognition of SARS-CoV-2. Signal Transduct. Target Ther..

[34] Huang, B. et al. Neutralization of SARS-CoV-2 VOC 501Y.V2 by human antisera elicited by both inactivated BBIBP-CorV and recombinant dimeric RBD ZF2001 vaccines. *bioRxiv*10.1101/2021.02.01.429069 (2021).

[35] Chen RE (2021). Resistance of SARS-CoV-2 variants to neutralization by monoclonal and serum-derived polyclonal antibodies. Nat. Med..

[36] Xie X (2021). Neutralization of SARS-CoV-2 spike 69/70 deletion, E484K and N501Y variants by BNT162b2 vaccine-elicited sera. Nat. Med..

[37] Liu Y (2021). Neutralizing activity of BNT162b2-elicited serum. N. Engl. J. Med..

[38] Liu Y (2021). BNT162b2-elicited neutralization against new SARS-CoV-2 spike variants. N. Engl. J. Med..

[39] Liu C (2021). Reduced neutralization of SARS-CoV-2 B.1.617 by vaccine and convalescent serum. Cell.

[40] Planas D (2021). Reduced sensitivity of SARS-CoV-2 variant Delta to antibody neutralization. Nature.

[41] Liu J (2021). BNT162b2-elicited neutralization of B.1.617 and other SARS-CoV-2 variants. Nature.

[42] Ewer KJ (2021). T cell and antibody responses induced by a single dose of ChAdOx1 nCoV-19 (AZD1222) vaccine in a phase 1/2 clinical trial. Nat. Med..

[43] Hassan AO (2020). A single-dose intranasal ChAd vaccine protects upper and lower respiratory tracts against SARS-CoV-2. Cell.

[44] Tauzin A (2021). A single dose of the SARS-CoV-2 vaccine BNT162b2 elicits Fc-mediated antibody effector functions and T cell responses. Cell Host Microbe.

[45] Voysey M (2021). Single-dose administration and the influence of the timing of the booster dose on immunogenicity and efficacy of ChAdOx1 nCoV-19 (AZD1222) vaccine: a pooled analysis of four randomised trials. Lancet.

[46] Ella R (2021). Safety and immunogenicity of an inactivated SARS-CoV-2 vaccine, BBV152: a double-blind, randomised, phase 1 trial. Lancet Infect. Dis..

[47] Richmond P (2021). Safety and immunogenicity of S-Trimer (SCB-2019), a protein subunit vaccine candidate for COVID-19 in healthy adults: a phase 1, randomised, double-blind, placebo-controlled trial. Lancet.

[48] Yang S (2021). Safety and immunogenicity of a recombinant tandem-repeat dimeric RBD protein vaccine against COVID-19 in adults: pooled analysis of two randomized, double-blind, placebo-controlled, phase 1 and 2 trials. Lancet Infect. Dis..

[49] Saad-Roy CM (2021). Epidemiological and evolutionary considerations of SARS-CoV-2 vaccine dosing regimes. Science.

