# Supplementary material for: RBD-homodimer, a COVID-19 subunit vaccine candidate, elicits immunogenicity and protection in rodents and nonhuman primates
Source: Cell Discov. 2021 Sep 7;7:82. doi: 10.1038/s41421-021-00320-y (PMC8423076; doi:10.1038/s41421-021-00320-y)
Supplement: Supplementary file 1 — Supplementary Information [file 41421_2021_320_MOESM1_ESM.pdf]

RBD-homodimer, a COVID-19 subunit vaccine candidate, elicits immunogenicity and protection in rodents and nonhuman primates

Xiaoyan Pan[1,3,4,#], Jian Shi[2,#], Xue Hu[1,3,#], Yan Wu[1], Liang Zeng[2], Yanfeng Yao[3], Weijuan Shang[1], Kunpeng Liu[1,4], Ge Gao[3], Weiwei Guo[1,4], Yun Peng[3], Shaohong Chen[1], Xiaoxiao Gao[3], Cheng Peng[3], Juhong Rao[1,4], Jiaxuan Zhao[1,4], Cheng Gong[2], Hui Zhou[2], Yudong Lu[2], Zili Wang[2], Xiliang Hu[2], WenJuan Cong[2], Lijuan Fang[2], Yongxiang Yan[2], Jing Zhang[2], Hui Xiong[2], Jizu Yi[2], Zhiming Yuan[1,3,4,*], Pengfei Zhou[2,*], Chao Shan[1,3,4,*], Gengfu Xiao[1,3,4,*]

[1]State Key Laboratory of Virology, Wuhan Institute of Virology, Chinese Academy of Sciences, Wuhan, China.

[2]Wuhan YZY Biopharma Co., Ltd, Wuhan, China.

[3]Center for Biosafety Mega-Science, Wuhan Institute of Virology, Chinese Academy of Sciences, Wuhan, China.

[4]University of the Chinese Academy of Sciences, Beijing, China.

[#]These authors contributed equally to this paper.

**To whom correspondence should be addressed: Gengfu Xiao, xiaogf@wh.iov.cn; Chao Shan, shanchao@wh.iov.cn; Pengfei Zhou, pfzhou@yzybio.com; Zhiming Yuan, yzm@wh.iov.cn.*

Supplemental materials

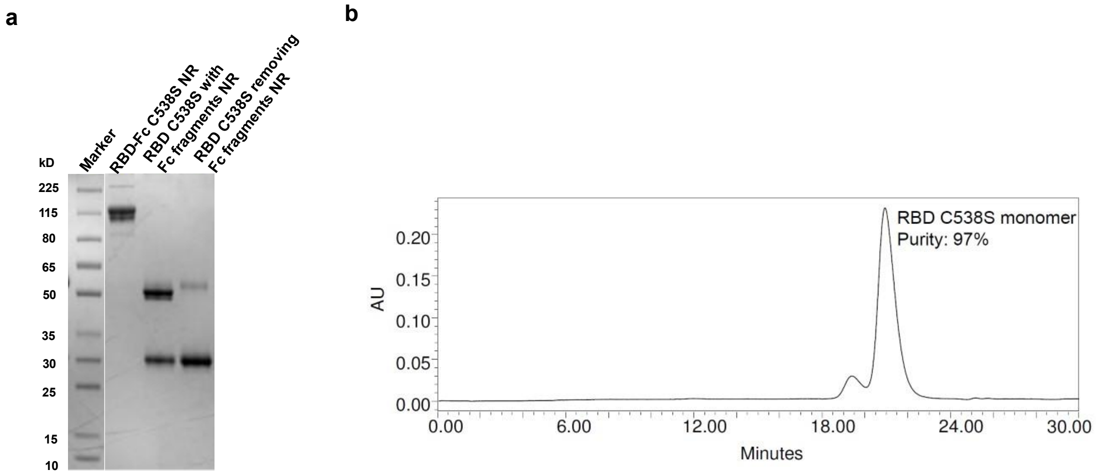

Supplementary Fig. S1 The formation of intermolecular disulfide bond affects the yield of RBD dimer.

a Analysis of the nonreduced RBD-C538S protein through SDS-PAGE and Coomassie brilliant blue staining. Lane 1: Marker; lane 2: RBD-Fc C538S; lane 3: RBD-Fc C538S digested with thrombin; lane 4: RBD-Fc C538S digested with thrombin and removing Fc fragments with Protein A affinity chromatography. RBD C to S was acquired from thrombin digestion on RBD-Fc and removing Fc fragments. **b** Analysis of RBD C538S by size exclusion chromatography (SEC), and the corresponding purities were marked in the graph.

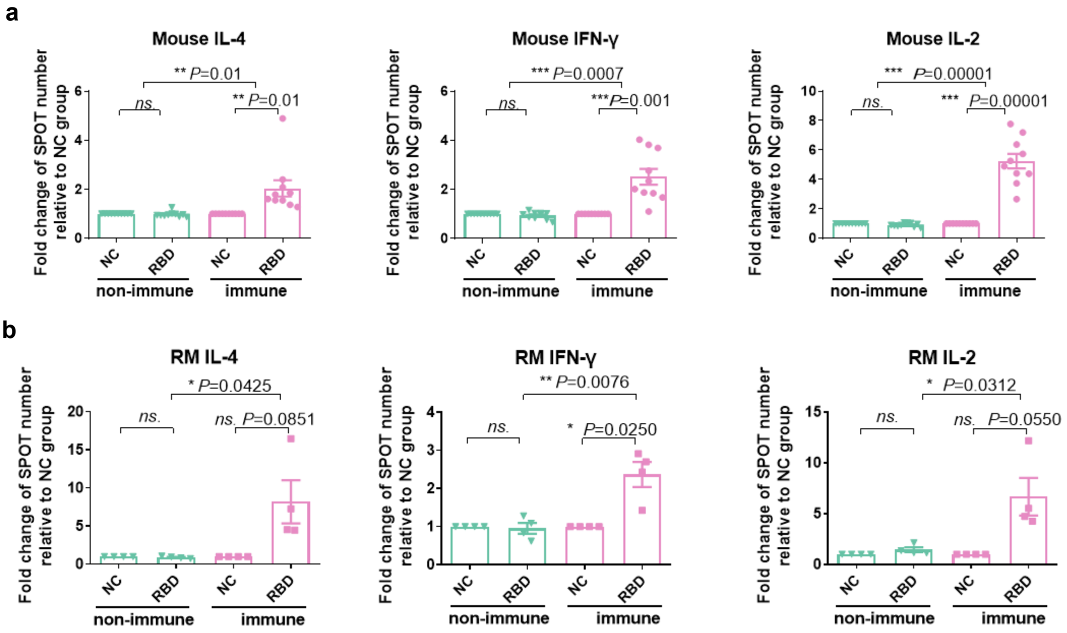

Supplementary Fig. S2 Cellular immune responses of RBD dimer vaccine in mice and rhesus macaques.

Splenic lymphocytes from mice (n=10) vaccinated with 5 μg RBD-AL (**a**) or PBMCs from RMs (n=4) vaccinated with 25 μg or 50 μg RBD-AL (**b**) were stimulated with RBD for 36 hours, and cytokines were detected by ELISA. NC, negative control. Data are Mean±SEM. *P* values were determined by unpaired Student's T-tests. ns. $P>0.05$, * $P<0.05$, ** $P<0.01$, *** $P<0.001$.

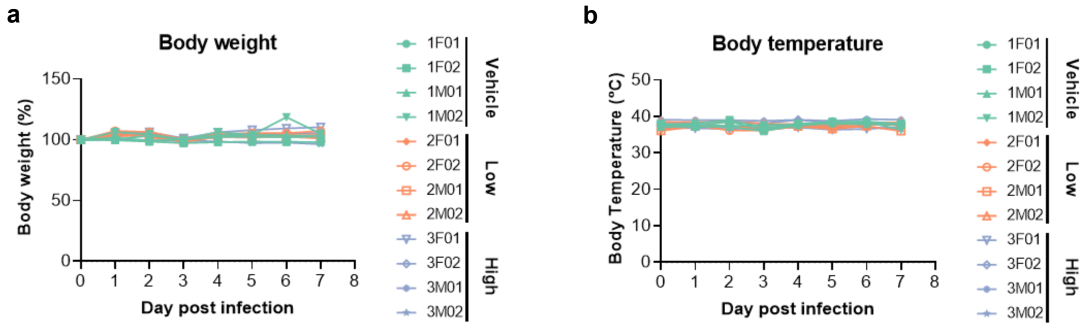

Supplementary Fig. S3 The body weight and body temperature of RMs after challenge.

Immunized RMs from vehicle, low dose (25 μg RBD) and high dose (50 μg RBD) group were challenged with SARS-CoV-2. Their body weight (**a**) and body temperature (**b**) were monitored lasting the duration of the challenge.

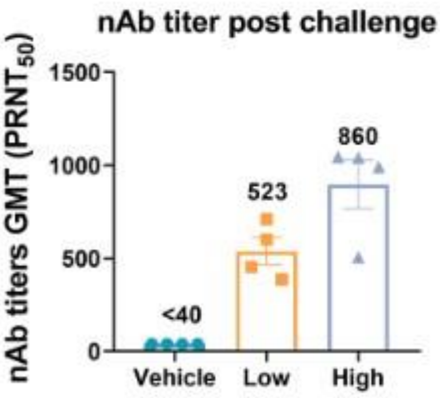

Supplementary Fig. S4 Neutralizing antibody titers of RM sera post challenge.

Sera post challenge (Day 49) were collected from RMs for neutralizing antibody titer detection, the GMTs of $PRNT_{50}$ from Vehicle, Low dose, and High dose group were calculated, and presented on the top of each column.

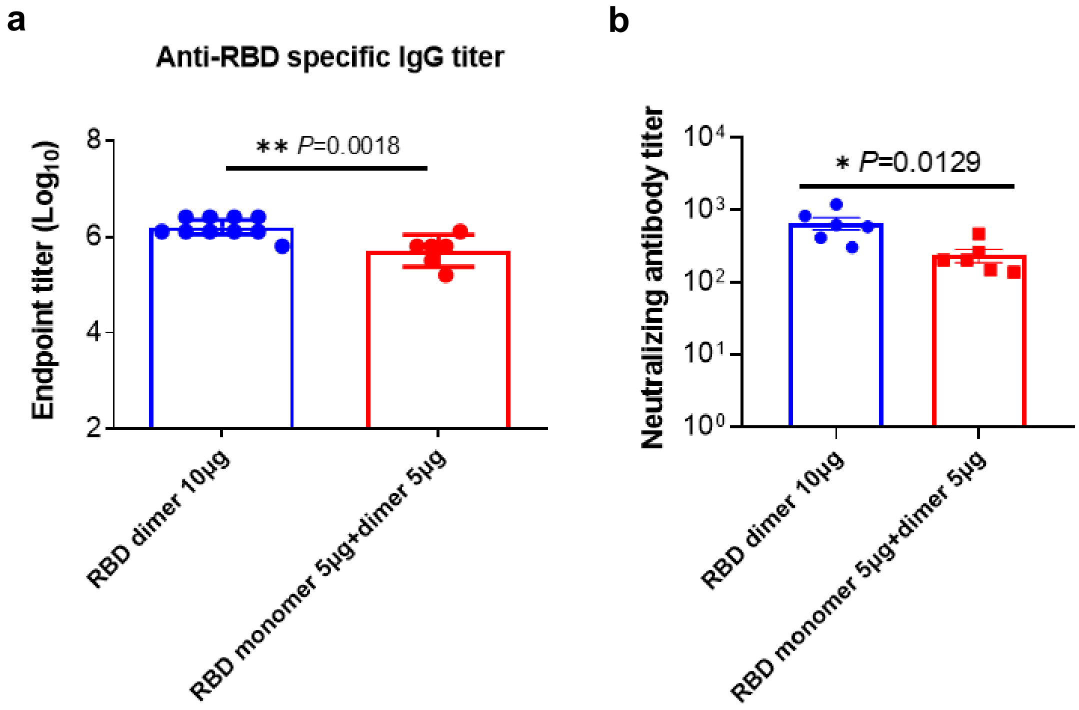

Supplementary Fig. S5 Comparison the immunogenicity of RBD dimer and monomer in mice.

Female BALB/c mice aged 6-8 weeks (n=6) were immunized with RBD dimer (10 μg) or a mixture of RBD dimer (5 μg) and monomer (5 μg) with AL via intramuscular route (i.m.) for three times with two-week intervals. Serum samples were collected from the ophthalmic vein 7 days after the third immunization for RBD-specific antibody detection by ELISA (**a**) and neutralizing antibody titer detection by hACE2-RBD blocking assays (**b**). * $P<0.05$, ** $P<0.01$.
